# Work functioning among young adults: the role of mental health problems from childhood to young adulthood

**DOI:** 10.1136/oemed-2021-107819

**Published:** 2021-10-28

**Authors:** Samira de Groot, Karin Veldman, Benjamin C Amick III, Ute Bültmann

**Affiliations:** 1 Department of Health Sciences, Community and Occupational Medicine, University Medical Center Groningen, University of Groningen, Groningen, The Netherlands; 2 Fay W. Boozman College of Public Health, University of Arkansas for Medical Sciences, Little Rock, Arkansas, USA

**Keywords:** occupational health, mental health, longitudinal studies, epidemiology

## Abstract

**Objectives:**

Mental health problems (MHPs) during childhood and adolescence are negatively associated with having a paid job in young adulthood. Yet, little is known about how young adults function at work, that is, do they experience difficulties in meeting their job demands given their health state. This longitudinal study aims to examine the impact of MHPs from childhood to young adulthood on young adults’ work functioning (WF).

**Methods:**

Data were used from 1004 participants in the TRacking Adolescents’ Individual Lives Survey, a Dutch prospective cohort study with 18-year follow-up. MHP trajectories, including 11, 13.5, 16, 19, 22 and 26 age points, were identified using growth mixture models. WF was assessed at age 29 with the Work Role Functioning Questionnaire 2.0 (WRFQ). Regression analyses were conducted to examine the association between MHP trajectories and WF.

**Results:**

Young adults with high-stable trajectories of internalising and externalising problems reported lower WF (mean WRFQ scores of 70.5 and 70.7, respectively) than those with low-stable trajectories (78.4 and 77.2), that is, they experience difficulties in meeting the work demands for more than one work day per full-time work week. Young adults with moderate-stable or decreasing MHP trajectories reported lower WF scores compared with those with low-stable trajectories.

**Conclusions:**

Both persistent high and elevated levels of MHPs from childhood to young adulthood are associated with lower WF scores in young adulthood compared with low-level MHPs. Occupational healthcare professionals should support young workers with a history of MHPs to optimise their work functioning.

**Key messages**

**What is already known about this subject?**

- Young adults with a history of mental health problems have an increased risk of not having a paid job.
- Among working young adults little is known how they are functioning during the workday.
- Among young adults at work, it is unclear whether and how a history of mental health problems is associated with their work functioning.
- Among working adults of all ages, mental health problems are associated with lower work functioning.

**What are the new findings?**

- Young adults with persistent high or elevated levels of mental health problems from childhood to young adulthood reported lower work functioning scores compared with their peers with persistent low levels of mental health problems.
- Young adults with persistently high-stable trajectories of mental health problems from childhood to young adulthood experience difficulties in meeting their work demands, approximately 1 day a week given a full-time work week.

**How might this impact on policy or clinical practice in the foreseeable future**

- Occupational healthcare policy and practice should focus on mental health problems from childhood to young adulthood to support a more inclusive labour market.
- Occupational healthcare professionals should support young adults with a history of mental health problems to optimise their work functioning.

## Introduction

Recent research shows that mental health problems in childhood and adolescence have a negative impact on young adult employment status (eg, having a paid job or not).^1–8^ Previous studies mainly examined mental health problems in relation to young adults’ employment status. Only a few examined the relationship between young adults with long duration mental health problems and employment status.^5 7^ Yet, little is known about how young adults with a job function at work and to what extent childhood and adolescent mental health problems affect their work functioning later in life. Work functioning, a concept at the intersection of a persons’ health and work performance, reflects the ability of a person to meet work demands given a physical or mental health state.^9–11^ As such, work functioning goes beyond the simple dichotomy of working or not working by assessing experiences in the work role.^9 10 12^

Prior research on mental health problems and work functioning has focused on adult working populations.^13–15^ Recent Dutch research has shown that work functioning is still impaired up to 1 year among workers who returned to work from being absent due to common mental disorders.^13^ US research among workers with a depressive disorder has shown that work limitations increase as the severity of depressive symptoms increases, that is, workers with a depression were twice as likely to be limited in their work as healthy workers.^14^ Even when depressive symptoms improved, US workers remained limited in their work functioning compared with relatively healthy workers.^15^ These studies lack a life-course perspective that considers the advantages and disadvantages of mental health experiences in childhood and adolescence a young worker brings in to the labour market and how these mental health experiences may affect work functioning in young adulthood.^16^ Earlier research conceptualised and measured mental health problems as health experience(s) during working adults’ life and not across the life course, that is, research did not link early life mental health experiences with later life work functioning. Longitudinal assessments using trajectories that capture the timing, duration and change over time of childhood and adolescent mental health problems should expand our understanding of how early life experiences affect later working life outcomes.

When entering the world of work, mental health problems developed in childhood and adolescence will probably shape the career and work functioning of young adults. Mental health problems in childhood and adolescence affect education, which affects labour market opportunities and work functioning or, if the mental health problems accumulate and carry forward into the labour market they can affect work functioning.^17^ A better understanding of the drivers of work functioning among young adults is important, because it may inform (occupational) healthcare professionals to guide and support young working adults, especially when young workers are affected by a history of mental health problems.

The aim of this study was to address the following question: ‘What is the impact of mental health problems from childhood to young adulthood (ages 11–26) on young adults’ work functioning at age 29?’. It is hypothesised that young adults’ work functioning is lowest among those with high stable levels of mental health problems from childhood to young adulthood, compared with young adults with low stable levels of mental health problems. The most recent 2019/2020 data from the 18-year follow-up TRacking Adolescents’ Individual Lives Survey (TRAILS) cohort study offer a unique opportunity to investigate work functioning among today’s young working adults.

## Methods

### Study design and sample

**Figure 1:**
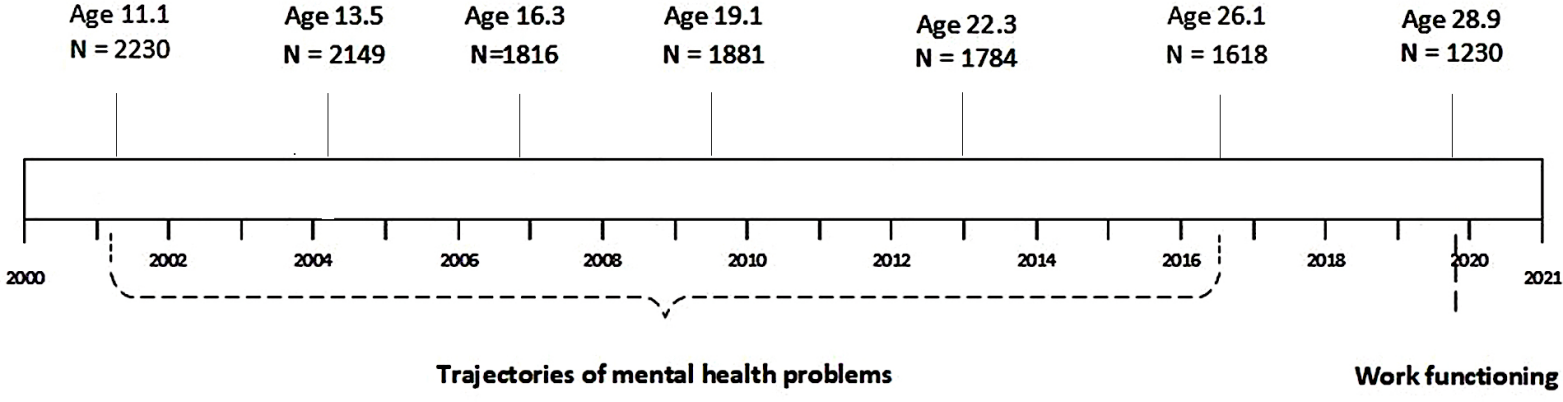
Timeline of TRAILS with mental health problems and work functioning.

Data were used from TRAILS, a prospective population-based cohort study that followed children from age 11 over a period of 18 years with seven follow-up waves.^18 19^ In March 2001, five municipalities from the three Northern provinces in the Netherlands provided information about all children born between 1 October 1989 and 30 September 1991. At baseline, 2230 children participated (76.0% from the initial sample) with a mean age of 11.1 years (SD=0.55). The children and one or both parents provided informed consent and the Dutch Central Committee on Research Involving Human Subjects approved all study protocols. In the second wave, children were on average 13.5 years (SD=0.53, N=2149, response rate 96.4%), third wave 16.3 years (SD=0.69, N=1816, 81.4%), fourth wave 19.1 years (SD=0.58, N=1881, 84.3%), fifth wave 22.3 years (SD=0.65, N=1775, 79.6%), sixth wave 25.7 years (SD=0.60, N=1618, 72.6%) and seventh wave 28.9 years (SD=0.60, N=1230, 55.2%). A graphical representation of the follow-up process of TRAILS is shown in figure 1. More detailed information about TRAILS can be found elsewhere.^18–21^ The final study sample consisted of N=1004 (81.6% of N=1230) young adults with a paid job at age 29, who provided information about their work functioning. Of the excluded participants, 64.6% did not have a paid job at age 29, 22.6% had a paid job but had missing data on work functioning, and 12.8% had missing data on both having a paid job and work functioning

### Measures

Work role functioning at age 29 was measured with the validated Work Role Functioning Questionnaire 2.0 10-item short version (WRFQ).^10 22 23^ The WRFQ measures the extent to which physical and mental health problems affect the ability to perform the work role in the past 4 weeks for a broad variety of work demands.^23^ The questionnaire is divided into four work demands subscales: ‘work scheduling and output demands’, ‘physical demands’, ‘mental and social demands’ and ‘flexibility demands’ (see online supplemental appendix table A1). Young adults respond on a five-point scale, 0=difficult all the time (100%), 1=difficult most of the time, 2=difficult half of the time (50%), 3=difficult some of the time) and 4=difficult none of the time (0%); and could also answer ‘Does not apply to my job’. The total WRFQ score and subscale scores were calculated by adding the items for each (sub)scale, divided by the corresponding number of completed items and multiplied by 25 to obtain scores between 0 and 100.^10^ If respondents endorsed ‘Does not apply to my job’, they were assigned the same score as ‘difficult none of the time (0%)’. If more than 20% of the items was missing, the total scale or subscale score was set to missing. More information about the psychometric properties of the total and subscale scores is provided in online supplemental appendix table A1. A higher WRFQ score indicates better work functioning. For example, a scale score of 75 indicates a young worker can meet the demands of his or her job 75% of the workday or workweek due to their physical health or emotional problems. Conversely, it means he or she cannot meet the job demands a quarter of the time due to their health status.

10.1136/oemed-2021-107819.supp1 Supplementary data

Mental health problems were assessed at ages 11, 13.5 and 16 by means of the youth self-report (YSR) and at ages 19, 22 and 26 with the adult self-report (ASR).^24^ Both the YSR and ASR are valid and reliable in measuring mental health problems in the past 6 months on a three-point Likert-scale: 0=not true, 1=somewhat or sometimes true, 2=very true or often true.^25 26^ Standardised scores were used for internalising problems (ie, anxious/ depressed, withdrawn/depressed and somatic complaints) and externalising problems (ie, aggressive, delinquent and intrusive behaviours). A higher score indicates higher levels of internalising or externalising problems.

Four covariates were selected based on the literature: sex, age, physical health and young adults’ educational level. Young adults’ age was their age at each measurement wave. Young adults’ physical health was measured at age 26 on a four-point Likert-scale ranging from 1=bad to 4=good. Young adults’ educational level was measured at age 29. Educational level was categorised into low (primary, lower vocational and lower secondary education), medium (intermediate vocational and intermediate secondary education) and high (higher secondary, higher vocational education and university).

To describe the study sample at age 29, information was included on type of employment (‘employee’, ‘self-employed’ and ‘both employee and self-employed’); type of employment contract (permanent contract, temporary contract) and work hours per week. Work hours were categorised as: ‘less than 20 hours’, ‘20–35 hours’ and ‘more than 35 hours’.

### Statistical analyses

First, the sample of young adults was described based on their sociodemographic characteristics and history of mental health problems. Second, in an attrition analysis, participants who participated in the sixth measurement wave (retention rate of 72.6%) and participants who dropped out on the seventh measurement wave (retention rate of 55.2%) were compared on sociodemographic characteristics and mental health problems. Third, to identify trajectories of mental health problems from age 11 to 26, growth mixture modelling was conducted.^27 28^ This method identifies subpopulations and longitudinal change within these subpopulations. Missing data were assumed to be missing at random. Each trajectory includes a random intercept and an unstructured time trend per individual (discrete time). The random intercept allows variation around the mean for individual trajectories. The time trend effect, the random intercept variances and the residual variances were estimated per class. Models were estimated with maximum-likelihood and robust SEs, and we used 500 random starting values per model. To identify the best model fit, several models were tested. Model selection was based on the optimal combination of different model fit indices, the entropy and substantive meaningfulness of classes. For model fit indices, the log-likelihood and the Bayesian information criteria (BIC) were used, with higher values of log-likelihood and lower values of BIC indicating better fit.^27 29^ The entropy refers to the precision of the assignment of individuals to the classes, and an entropy of 0.6 or above indicates better separation and more certainty in classification.^30^ Fourth, we performed linear regression analyses to examine the association between class membership of mental health problem trajectories and work functioning. For all regression analyses, we calculated crude associations (model 1), adjusted for age and sex (model 2) and additionally adjusted for young adults’ physical health and educational level (model 3). Models were estimated for the total work functioning score and each subscale score separately. Only the results of the total score of work functioning is presented (table 2). The results of the subscales of work functioning are shown as online supplemental table S1. Fifth, a sensitivity analysis with the WRFQ answer category ‘Does not apply to my job’ treated as a missing value according to the original scoring method was conducted to compare with the main analysis results. The statistical analyses were performed in SPSS V.26.0 and Mplus V.8.5.

10.1136/oemed-2021-107819.supp2 Supplementary data

## Results

### Sample characteristics

Most young adults (average age 28.9 years) were female (61.6%), highly educated (51.5%), worked as an employee (87.2%) with a permanent contract (62.4%) for more than 35 hours per week (56.5%) and reported moderate to good physical health (85.9%). The attrition analysis showed that non-participants in the seventh measurement wave were more likely to be male, low educated, unemployed and/or having high levels of externalising problems at the sixth measurement wave. Young adults reported a mean total work functioning score of 85.5 (SD 16.3), which translates into experiencing difficulties meeting work demands, on average 14.5% of their working time. Each subscale varied, with physical demands the highest functioning score (90.3, SD 18.0) and mental and social demands the lowest (81.3, SD 22.6) (table 1).

**Table 1:** Sample description for the total sample and by mental health trajectories

	Age*	Total sample	Internalising problems				Externalising problems			
		(N=1004)	High-stable (N=225)	Moderate-stable (N=230)	Decreasing (N=293)	Low-stable (N=256)	High-stable (N=193)	Moderate-stable (N=292)	Decreasing (N=282)	Low-stable (N=237)
Outcome variable										
Work role functioning (mean, SD)	29									
Total score (N=1004)		85.5 (16.3)	80.5 (17.1)	86.2 (14.4)	86.2 (16.2)	88.5 (16.5)	81.9 (16.6)	84.0 (17.5)	86.2 (15.9)	89.5 (14.1)
WSOD (N=1003)		86.1 (18.1)	80.4 (20.0)	86.0 (17.4)	87.3 (17.6)	89.7 (16.4)	81.6 (20.1)	84.7 (19.6)	87.3 (16.7)	89.9 (15.1)
PD (N=1004)		90.3 (18.0)	86.3 (19.4)	91.3 (17.2)	91.2 (17.8)	92.0 (17.2)	88.7 (19.0)	88.7 (20.0)	90.7 (17.4)	93.5 (14.5)
MSD (N=1004)		81.3 (22.6)	76.0 (24.0)	81.3 (21.3)	82.0 (21.8)	85.0 (22.6)	78.1 (23.6)	79.1 (23.7)	81.4 (22.4)	86.3 (19.7)
FD (N=1004)		81.7 (23.8)	76.3 (26.4)	83.8 (21.1)	81.4 (23.6)	85.0 (23.3)	77.0 (25.6)	81.0 (24.2)	82.1 (23.4)	85.9 (21.4)
Background variables										
Sex (N, %)	11									
Male		386 (38.4)	49 (21.3)	92 (40.0)	91 (31.1)	154 (60.2)	83 (43.0)	102 (34.9)	116 (41.1)	85 (35.9)
Female		618 (61.6)	176 (78.2)	138 (60.0)	202 (68.9)	102 (39.8)	110 (57.0)	190 (65.1)	166 (58.9)	152 (64.1)
Age (mean, SD)	29	28.9 (0.6)	28.9 (0.6)	29.0 (0.6)	28.8 (0.6)	29.0 (0.6)	28.8 (0.6)	28.9 (0.6)	28.9 (0.6)	29.0 (0.57)
Physical health (mean, SD)†	26	3.2 (0.8)	2.9 (0.8)	3.2 (0.7)	3.3 (0.7)	3.5 (0.7)	3.1 (0.7)	3.1 (0.8)	3.3 (0.8)	3.4 (0.7)
Educational level (N, %)	29									
Low		90 (9.0)	25 (11.1)	12 (5.2)	27 (9.2)	26 (10.2)	26 (13.5)	29 (9.9)	19 (6.7)	16 (6.8)
Medium		397 (39.5)	102 (45.3)	86 (37.4)	124 (42.3)	85 (33.2)	87 (45.1)	101 (34.6)	125 (44.3)	84 (35.4)
High		517 (51.5)	98 (43.6)	132 (57.4)	142 (48.5)	145 (56.6)	80 (41.5)	162 (55.5)	138 (48.9)	137 (57.8)

*Age at which variable was measured.

†Physical health scores range from 1 to 4.

FD, flexibility demands; MSD, mental and social demands; PD, physical demands; WSOD, work scheduling and output demands.

### Trajectories of mental health problems from childhood to young adulthood

**Figure 2:**
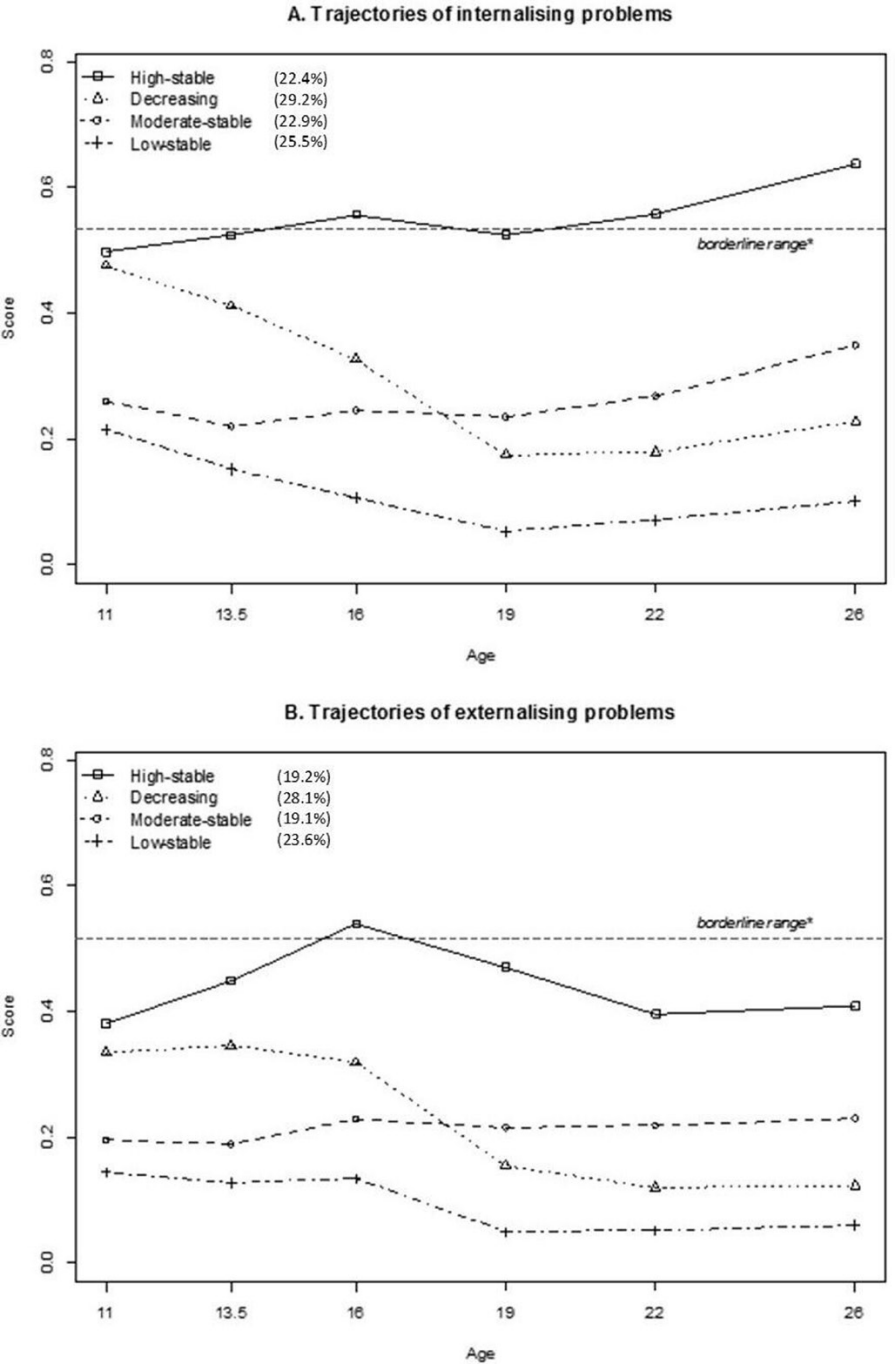
(A, B): Young adults classified according to their trajectory of YSR/ASR internalising and externalising problems from ages 11–26 years. *Borderline range is based on ASEBA’s cut-off scores for the Netherlands.^39^ ASR, adult self-report; YSR, youth self-report.

For both internalising and externalising problems, the differences in fit statistics were minimal for the four-class and five-class model (online supplemental table S2). Therefore, the substantive meaningfulness and interpretability of the classes were the deciding factors to prefer the four-class model above the five-class model. Young adults were assigned to one of the four trajectories based on their posterior probabilities ranging from 0.72 to 0.89. The high-stable trajectory includes young adults with persistent high scores of internalising (N=225; 22.4%) and externalising problems (N=193; 19.2%). The decreasing trajectory represents young adults with decreasing scores of internalising (N=293; 29.2%) and externalising problems (N=282; 28.1%). The moderate-stable trajectory consists of young adults with persistent moderate scores of internalising (N=230; 22.9%) and externalising problems (N=292; 29.1%). The low-stable trajectory represents young adults with persistent low scores of internalising (N=256; 25.5%) and externalising problems (N=237; 23.6%)(figure 2).

### Trajectories of mental health problems (age 11–26) and total work functioning (age 29)

Compared with young adults in a low-stable trajectory of mental health problems, mental health problem trajectories from childhood to young adulthood of all other young adults were negatively associated with total work functioning (table 2). With a score of 70.5 (subtracting the beta coefficient (−7.9) from the corresponding intercept (78.4)), the total work functioning score of young adults in a high-stable trajectory of internalising problems was most impacted. This means that they could not meet their work demands for 29.5% of their working time, that is more than 1 day in a full-time work week. Young adults in a decreasing trajectory or moderate-stable trajectory of internalising problems scored, respectively, 75.5 and 75.9 (n.s.) on the total work functioning scale. Similar results were found for externalising problems, that is, young adults in a high-stable trajectory scored 70.7 on the total work functioning scale, compared with scores of 72.5 and 74.3 for young adults in a moderate-stable and decreasing trajectory, respectively.

**Table 2:** Regression analyses of trajectories of internalising and externalising problems from age 11 to 26 and total score of work functioning at age 29

	Model 1		Model 2		Model 3	
	Beta	SE	Beta	SE	Beta	SE
Intercept	88.5***	1.0	89.8***	1.2	78.4***	3.1
Internalising problems (ref. low-stable)						
High stable	−8.0***	1.5	−9.0***	1.5	−7.9***	1.6
Decreasing	−2.3	1.4	−3.1*	1.4	−2.9*	2.9
Moderate stable	−2.3	1.5	−2.7	1.5	−2.5	1.5
Age (0=mean)			−1.6	0.9	−1.8*	0.9
Sex (ref. female)			−1.8	1.1	−1.7	1.1
Physical health					1.5*	0.7
Educational level (ref. low)						
Medium					6.0***	1.9
High					7.4***	1.8
Intercept	89.5***	1.1	89.5***	1.1	77.2***	3.0
Externalising problems (ref. low-stable)						
High stable	−7.6***	1.7	−7.7***	1.6	−6.5***	1.6
Decreasing	−3.3*	1.4	−3.3*	1.4	−2.9*	1.4
Moderate stable	−5.4***	1.4	−5.5***	1.4	−4.7***	1.4
Age (0=mean)			−1.4	0.9	−1.6	0.9
Sex (ref. female)			0.0	1.1	0.2	1.0
Physical health					1.8**	0.7
Educational level (ref. low)						
Medium					5.6**	1.9
High					7.1***	1.8

Model 1: crude | Model 2: adjusted for age (0 = mean) and sex (ref. female) | Model 3: Model 2 + physical health and educational level (ref. low)

*P*≤*0.05, **p*≤*0.01, ***p*≤*0.001.

### Trajectories of mental health problems and subscales of work functioning

Regarding internalising problems, compared with young adults in a low-stable trajectory, a high-stable trajectory of internalising problems was negatively associated with all subscales of work functioning (online supplemental table S1). Young adults in a moderate-stable trajectory of internalising problems scored 78.7 on the work scheduling and output demands scale. Young adults in a decreasing trajectory of internalising problems had difficulties to meet the work demands in all subscales, except flexibility demands. Regarding externalising problems, compared with young adults in a low-stable trajectory, young adults in a high-stable or moderate-stable trajectory had difficulties to meet the work demands in all subscales. Young adults in a decreasing trajectory of externalising problems scored 69.0 on the mental and social demands scale.

### Sensitivity analysis

The sensitivity analysis, in which the sample for whom work functioning was calculated according to the original scoring method (N=914) was compared with the sample with the answer category ‘Does not apply to my job’ treated similar as ‘Difficult none of the time’ (N=1004), revealed only minor differences with the results of the main analysis (online supplemental table S3).

## Discussion

This study went beyond the dichotomy of working or not working by providing in-depth information about how young adults were functioning on their job and took their history of mental health problems into account. To the best of our knowledge this study is the first to (add a life-course perspective to) investigate mental health problems from childhood to young adulthood and work functioning in young adulthood. With unique 18-year follow-up data, we examined the impact of mental health problems from childhood to young adulthood (ages 11–26) on young adults’ work functioning at age 29. Four trajectories were identified for both internalising and externalising problems: a high-stable, moderate-stable, decreasing and low-stable trajectory. For internalising problems 22.4% and for externalising problems 19.2% of the participants was assigned to the high-stable trajectory group, which corresponds to 20%–25% of young adults in OECD countries reporting mental health problems.^31^ We found that elevated levels of mental health problems in itself during childhood, adolescence or young adulthood were associated with lower work functioning at age 29. As hypothesised, especially young adults with persistently high levels of internalising or externalising problems experienced difficulties in meeting their work demands for approximately 29% of their working time. But also young adults with decreasing levels of internalising or externalising problems experienced difficulties in meeting their work demands. This means that even when young adults with a history of mental health problems have a job, their work functioning is substantially affected.

Our findings are in line with the existing literature, showing that mental health problems affect work functioning.^12–14^ However, a detailed comparison of our findings with previous studies is challenging because of different study samples and types of mental health problems (eg, depression^15^). While the previous studies on work functioning mainly focused on adult populations,^13–15^ the current study added new insights on work functioning of today’s young working adults. Young adults reported on average a total work functioning score of 85.5, which is comparable to the work functioning of the general working population in the Netherlands, that is, 84.2.^11^ Moreover, while existing literature lacks a more extended focus on childhood and adolescence mental health problems, our study introduced a life-course perspective to work functioning, and showed that it is important to take the history of mental health problems into account.

The finding of lower work functioning among young adults with elevated levels of internalising problems may be due to the negative effect of these internalising problems on cognitive and social functioning. Impaired cognitive and social functioning may induce fatigue, limited concentration and motivation, and disrupt interpersonal relationships,^32–34^ and are likely to negatively affect work functioning.^35^ Regarding externalising problems, previous research showed that in particular rule-breaking behaviour (as a subscale of externalising problems) in childhood, adolescence and young adulthood increased the risk of sickness absence in young adulthood.^36^ Furthermore, Roberts *et al*, showed that employees diagnosed with conduct disorder in childhood or adolescence more often reported counterproductive work behaviours, including disciplinary problems, theft, drug and alcohol abuse.^37^ These findings demonstrate the difficulties of being and staying at work for those who experience(d) mental health problems and highlight the necessity for this group to receive support from employers and occupational healthcare providers.

A strength of the current study is the use of the longitudinal TRAILS study with 18-year follow-up. Work functioning was assessed using the validated 10-item version of the WRFQ 2.0.^22^ Reliable measures were also used to measure internalising and externalising mental health problems, that is, the YSR and ASR.^25 26^ Multiple measurements of mental health problems made it possible to identify trajectories of internalising and externalising problems from childhood to young adulthood. The most recent 2019/2020 data from TRAILS study allowed us to be the first study with substantial follow-up that examined the relationship between a history of mental health problems and work functioning among young adults from a life-course perspective.

When interpreting the results, the following methodological issues must be considered. Though reasonable for an 18-year follow-up study among young adults, the lost to follow-up of 17% from the sixth to the seventh measurement wave may have biased the results. The attrition analysis demonstrated that non-participants were more likely to be male, low educated, unemployed and/or having high levels of externalising problems, suggesting that our findings may be an underestimation of the impact of a history of mental health problems on work functioning. Another issue concerns the potential measurement error in class membership assignment, as young adults may have been assigned to a different trajectory than they belong to. However, based on the average posterior probability that ranges from 72% to 89% between the trajectories, we conclude that the majority of the young adults are assigned to the correct trajectory, and measurement error is limited. In addition, when looking at the WRFQ physical demands subscale it is interesting to note that the moderate-stable and decreasing trajectories of internalising problems were not found to be different from the low-stable trajectory. The work functioning subscales of the short WRFQ, and in particular the physical health subscale, may not fully capture all work demands. Therefore, the results of the subscales should be interpreted with caution. Further research using the full WRFQ 2.0, is needed to examine the associations of mental health trajectories and work functioning subscales in more detail.

Our findings have several implications for policy and practice as well as for further research. It is of great importance to focus on the guidance and support of vulnerable young workers with a history of mental health problems, as the study results clearly show that their work functioning is affected. Especially now in the current COVID-19 pandemic and beyond, young workers may be disproportionately affected.^38^ With that said, policy-makers should prioritise providing better services for preventing mental health problems, not only for young workers, but especially for children and adolescents to ensure a healthy start of their working lives. For young workers, workplace interventions targeting both mental health problems and work outcomes, including work functioning, are needed to prevent more severe consequences, such as sickness absence and work disability. The WRFQ may be used by occupational physicians as instrument to identify the work demands, which are experienced as difficult, and may function as an indicator for potential work accommodations. Furthermore, for future research it is recommended to repeatedly measure work functioning over time to examine the potential bi-directional relationship between mental health problems and work functioning. More research is also needed to unravel the mechanisms linking mental health problems and work functioning. Also other factors may affect work functioning, such as work-related factors, for example, occupational classes or job duration, and the interplay between work and private life. Further research should also expand on contextual factors, for example, the potential mediating or moderating role of psychosocial work environment factors, such as interpersonal relationships at work, or educational attainment.

In conclusion, mental health problems during childhood, adolescence and young adulthood affect work functioning in young adulthood. Not only young adults with persistently high levels of mental health problems experience difficulties in meeting their job demands, also young adults with elevated levels of mental health problems are limited in their work functioning. These findings clearly support the relevance of applying a life-course perspective when investigating mental health problems and work functioning. Therefore, it is recommended for policy makers to focus on mental health problems from childhood to young adulthood to support a more inclusive labour market. For occupational physicians and healthcare professionals, it is recommended to support young adults with a history of mental health problems to optimise their work functioning.

## Data Availability

Data may be obtained from a third party and are not publicly available. TRAILS data of the T1, T2, T3, T4 and T5 measurement wavesare deposited in the Data Archiving and Networked Services of the Royal Dutch Academy of Sciences (DANS-KNAW) and access can be requested at “http://www.dans.knaw.nl".
