# Supplementary material for: Work functioning among young adults: the role of mental health problems from childhood to young adulthood
Source: Occup Environ Med. 2021 Oct 28;79(4):217–23. doi: 10.1136/oemed-2021-107819 (PMC8921561; doi:10.1136/oemed-2021-107819)
Supplement: Supplementary data [file oemed-2021-107819supp001.pdf]

Appendix

Table A1: WRFQ 2.0 sample description (N=1055)

	Valid N	Not applicable N (%)	Missing N (%)	Mean (SD)	Range	Cronbach's α*
**WSOD**	**899**	105 (10.0)	52 (4.9)	85.43 (18.31)	0-100	0.79
Start as soon as you arrived	**932**	72 (6.8)	51 (4.8)	86.88 (21.96)		
Work without mistakes	**946**	57 (5.4)	52 (4.9)	85.15 (21.82)		
Work fast enough	**934**	70 (6.6)	51 (4.8)	83.24 (23.01)		
**PD**	**724**	280 (26.5)	51 (4.8)	88.97 (19.76)	0-100	0.87
Stay in one position	**909**	95 (9.0)	51 (4.8)	86.96 (23.71)		
Repeat the same motions	**791**	213 (20.2)	51 (4.8)	88.53 (22.30)		
Bend, twist or reach	**793**	211 (20.0)	51 (4.8)	89.60 (22.10)		
**MSD**	**956**	48 (4.6)	51 (4.8)	80.57 (22.81)	0-100	0.82
Do work carefully	**961**	43 (4.1)	51 (4.8)	84.63 (24.41)		
Concentrate on work	**965**	39 (3.7)	51 (4.8)	76.30 (25.20)		
**FD**	**947**	57 (5.4)	51 (4.8)	80.78 (24.10)	0-100	0.88
Set priorities	**959**	45 (4.3)	51 (4.8)	80.68 (25.34)		
Perform multiple tasks	**956**	48 (4.5)	51 (4.8)	80.96 (25.73)		
**Total score**	**914**	89 (8.4)	52 (4.9)	84.30 (17.23)	2.50-100	0.91

Alphas calculated in SPSS (listwise deletion)
