# Supplementary material for: Work functioning among young adults: the role of mental health problems from childhood to young adulthood
Source: Occup Environ Med. 2021 Oct 28;79(4):217–23. doi: 10.1136/oemed-2021-107819 (PMC8921561; doi:10.1136/oemed-2021-107819)
Supplement: Supplementary data [file oemed-2021-107819supp002.pdf]

Supplemental material

Table S1: Regression analyses of trajectories of internalising and externalising problems from age 11 to age 26 on the subscales of work functioning at age 29:

	**Work Role Functioning**							
	WSOD		PD		MSD		FD	
	Beta	SE	Beta	SE	Beta	SE	Beta	SE
Intercept	**82.5*** **	**3.4**	**76.1*** **	**3.4**	**74.7*** **	**4.3**	**79.6*** **	**4.5**
Internalising problems (ref. low-stable)								
High-stable	**-9.5*** **	**1.8**	**-4.4***	**1.7**	**-9.2*** **	**2.2**	**-9.4*** **	**2.3**
Moderate-stable	**-3.8***	**1.7**	-0.8	1.6	-4.0	2.1	-1.7	2.2
Decreasing	**-3.2***	**1.6**	-0.6	1.5	**-3.9***	**2.0**	**-4.8***	**2.1**
Age (0 = mean)	-1.5	1.0	-0.9	0.9	**-2.6***	**1.2**	**-2.6***	**1.3**
Sex (ref. female)	-2.1	1.2	-0.1	1.2	-1.9	1.5	**-3.3***	**1.6**
Physical health	**1.6***	**0.8**	**1.5***	**0.8**	1.5	1.0	1.2	1.0
Educational level (ref. low)								
Medium	**4.1***	**2.1**	**9.2*** **	**2.0**	**7.0** **	**2.6**	3.0	2.8
High	3.2	2.1	**13.6*** **	**2.0**	**7.2** **	**2.6**	**4.7***	**2.7**
Intercept	**80.1*** **	**3.4**	**76.4*** **	**3.3**	**73.6*** **	**4.2**	**77.8*** **	**4.5**
Externalising problems (ref. low-stable)								
High-stable	**-7.6*** **	**1.8**	**-3.7***	**1.7**	**-7.3*** **	**2.2**	**-8.1*** **	**2.3**
Moderate-stable	**-4.5** **	**1.6**	**-3.9***	**1.5**	**-6.5*** **	**2.0**	**-4.5***	**2.1**
Decreasing	-2.4	1.6	-2.0	1.5	**-4.6***	**2.0**	-3.4	2.1
Age (0 = mean)	-1.4	1.0	-0.8	0.9	**-2.3***	**1.2**	-2.2	1.3
Sex (ref. female)	-0.3	1.2	0.6	1.1	-0.1	1.5	-1.2	1.5
Physical health	**2.1** **	**0.8**	**1.7***	**0.8**	**1.9***	**1.0**	1.5	1.0
Educational level (ref. low)								
Medium	3.5	2.1	**9.0*** **	**2.1**	**6.5***	**2.6**	2.7	2.8
High	2.7	2.1	**13.5*** **	**2.0**	**6.8** **	**2.6**	**4.5**	**2.7**

****: p=≤0.001 **:p=≤0.01 *:p=≤0.05 [a]: borderline significant at p=≤0.05*

WSOD: Work Scheduling and Output Demands; PD: Physical Demands; MSD: Mental and Social Demands; FD: Flexibility Demands

de Groot S, *et al. Occup Environ Med* 2022; 79:217–223. doi: 10.1136/oemed-2021-107819

Table S2: Trajectories of mental health problems from age 11 to 26: fit statistics of latent class growth models with increasing number of classes for internalising and externalising problems

	Class	Number of parameters	Log-likelihood	Bayes information criterion	Entropy
Internalising problems	3	41	2817.9	-5319.9	0.65
	**4**	**55**	**3002.4**	**-5580.9**	**0.62**
	5	69	3132.2	-5732.6	0.63
Externalising problems	3	41	4878.6	-9441.1	0.68
	**4**	**55**	**5135.5**	**-9847.1**	**0.64**
	5	69	5241.3	-9950.7	0.61

Note: used model is indicated in bold

Table S3: Regression analyses of trajectories of internalising and externalising problems from age 11 to age 26 on the total score of work functioning at age 29 with 'not applicable' considered as missing (N=914)

	**Total**	
	Beta	SE
Intercept	**75.3*** **	**3.5**
Internalising problems (ref. low- stable)		
High-stable	**-7.9*** **	**1.7**
Moderate-stable	-2.5	1.6
Decreasing	**-3.3***	**1.6**
Age (0 = mean)	-1.5	1.0
Sex (ref. female)	-1.2	1.2
Physical health	**1.9***	**0.8**
Educational level (ref. low)		
Medium	**6.2****	**2.1**
High	**7.7*** **	**2.1**
Intercept	**74.5*** **	**3.4**
Externalising problems (ref. low- stable)		
High-stable	**-6.5*** **	**1.8**
Moderate-stable	**-5.4*** **	**1.6**
Decreasing	**-3.6***	**1.6**
Age (0 = mean)	-1.3	0.9
Sex (ref. female)	0.6	1.2
Physical health	**2.2****	**0.8**
Educational level (ref. low)		
Medium	**5.9****	**2.2**
High	**7.6*** **	**2.1**

****: p=≤0.001 **:p=≤0.01 *:p=≤0.05*

2
